# Antiseptic Surgery—The Principles, Modes of Application and Results of the Lister Dressing

**Published:** 1886-10

**Authors:** 


ANTISEPTIC SURGERY—THE PRINCIPLES, MODES OF APPLICATION AND RESULTS OF THE LISTER DRESSING. By Just-Lucas Champeonniere, Surgeon to the Hospital Tenon, Member of the Societe de Chirurgie, editor of the Journal de Medecine et de Chirurgie Proliques. Translated from the second and completely revised edition with the special sanction of the author, and edited by Frederic Henry Gerrish, A. M., M. D., Surgeon to the Maine General Hospital, Professor of Materia and Therapeutics in Bowdoin College, etc. Portland: Loring, Short and Hardman. 1881.

The application of Listerism in surgery has not been so generally satisfactory as the fruits of this teaching in the adoption of absolute cleanliness throughout the treatment of wounds.

While the carbolic spray has been found in many instances prejudicial to patient and operator, the occlusion of wounds, which is a part of the process, notwithstanding the statement of our author to the contrary, fails in numerous cases to subserve so good a purpose as open dressings.

The general principle being recognized as correct, that all injuries to vital structures, whether from accidents or by operative procedures, are more disposed to union by first intention when the atmosphere is excluded from contact with the dissected tissues, it must still be conceded that in cases prone to develop suppurations, this result cannot be obviated always by the Lister dressing.

This work presents the method fairly and clearly as practiced when it was issued, but many new phases in the use of curative agencies by numerous practical operations in the different departments of surgery have been presented within the past five years, which are calculated to supersede the measures inculcated in this book. Whether the new era doctrine of bacterial etiology shall prevail generally throughout the medical profession, must depend not so much on further investigations with the microscope as upon the proper interpretation of biological phenomena involved in the physiological and pathological conditions of the organism.

This volume is well printed on good paper, with neat cloth binding, and reflects much credit upon the publishers.

